# Supplementary figures and images for: Noninvasive ventilation in critically ill very old patients with pneumonia: A multicenter retrospective cohort study
Source: PLoS One. 2021 Jan 27;16(1):e0246072. doi: 10.1371/journal.pone.0246072 (PMC7840033; doi:10.1371/journal.pone.0246072)

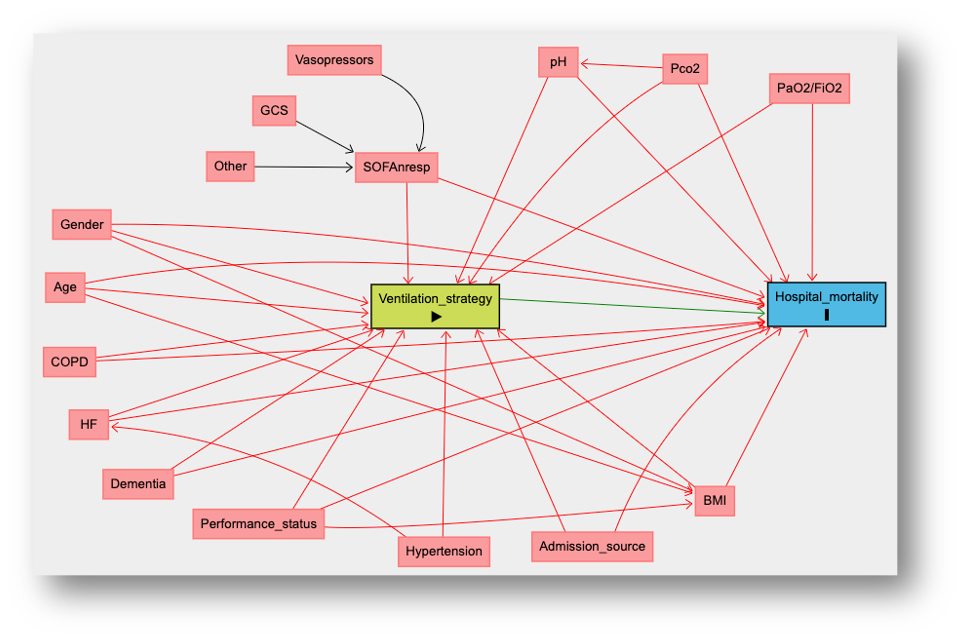

Supplement: S1 Fig — COPD: Chronic obstructive pulmonary disease; HF: Heart failure; BMI: Body mass index; SOFAnresp: non-respiratory SOFA score; GCS: Glasgow coma scale. * This directed acyclic graph was built with the online version of DAGitty. (TIF) [file pone.0246072.s002.tif]

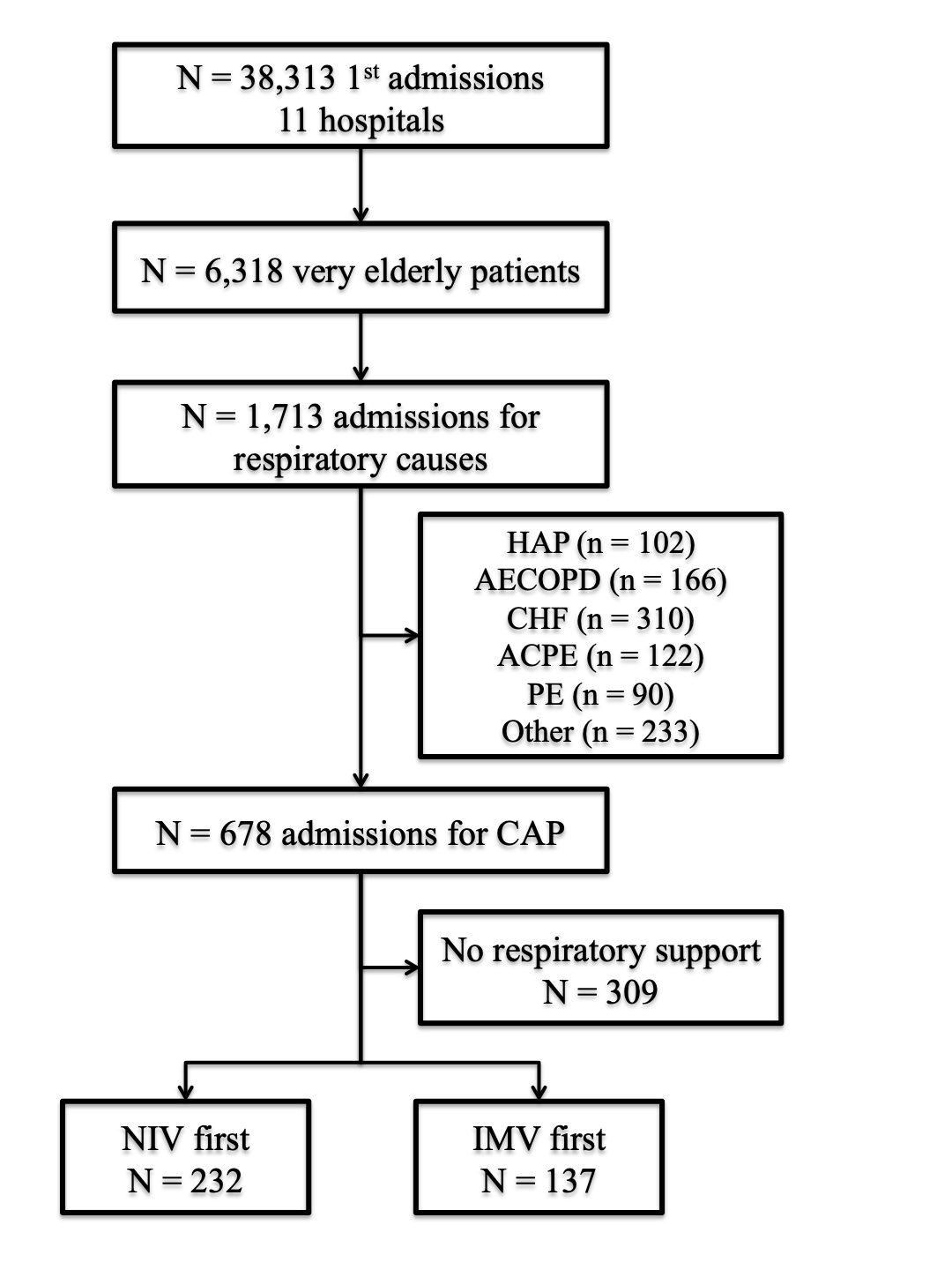

Supplement: S2 Fig — * Readmissions excluded from this flowchart. (TIF) [file pone.0246072.s003.tif]

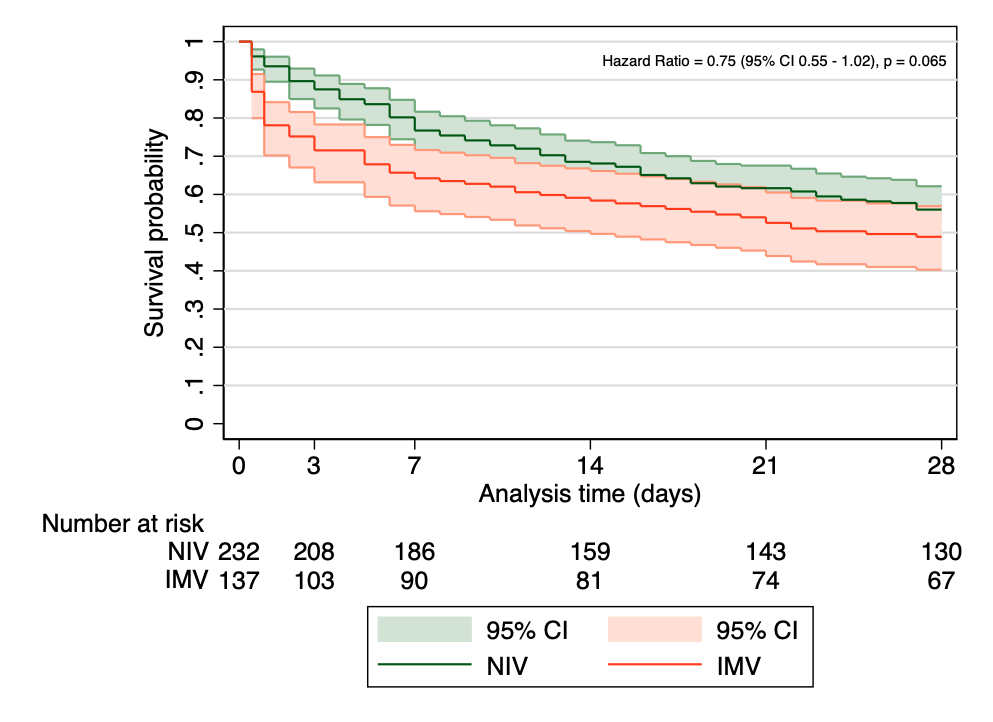

Supplement: S3 Fig — Hazard ratio from a Cox proportional hazards model without statistical adjustment. (TIF) [file pone.0246072.s004.tif]

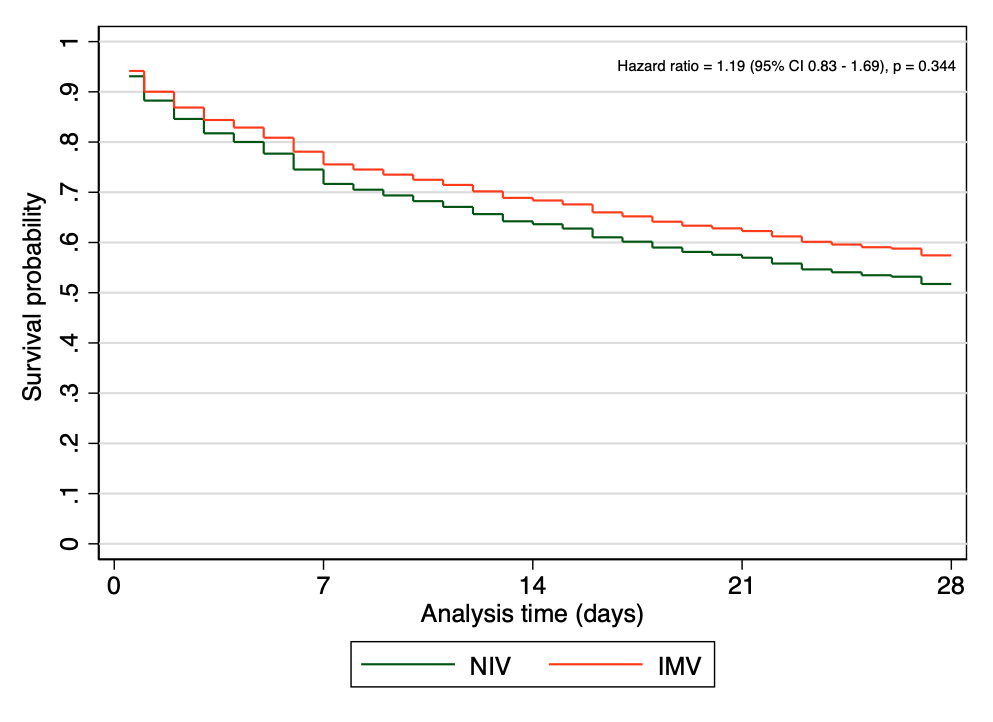

Supplement: S4 Fig — Hazard ratio from a Cox proportional hazards model adjusted for age, sex, SAPS3 score and modified frailty index (mFI). (TIF) [file pone.0246072.s005.tif]
