# Supplementary material for: Noninvasive ventilation in critically ill very old patients with pneumonia: A multicenter retrospective cohort study
Source: PLoS One. 2021 Jan 27;16(1):e0246072. doi: 10.1371/journal.pone.0246072 (PMC7840033; doi:10.1371/journal.pone.0246072)
Supplement: S1 Table — (DOCX) [file pone.0246072.s006.docx]

## S1 Table. Proportion of missing data among variables included in the model.

| **Variable** | **Number of patients with missing data (%)** |
| --- | --- |
| Non-respiratory SOFA | 31/369 (8.4%) |
| BMI | 117/369 (31.7%) |
| pH | 128/369 (34.7%) |
| P_a_co_2_ | 128/369 (34.7%) |
| P_a_o_2_/F_i_o_2_ | 161/369 (43.6%) |
| There was no missing data for the other variables included in the primary outcome analysis model | |
