# Supplementary material for: Noninvasive ventilation in critically ill very old patients with pneumonia: A multicenter retrospective cohort study
Source: PLoS One. 2021 Jan 27;16(1):e0246072. doi: 10.1371/journal.pone.0246072 (PMC7840033; doi:10.1371/journal.pone.0246072)
Supplement: S2 Table — (DOCX) [file pone.0246072.s007.docx]

## S2 Table. Sensitivity analyses for the primary outcome.

|  | **Odds ratio*** | **95% CI** | **p-value** |
| --- | --- | --- | --- |
| Final model ^a^ | 0.81 | 0.46 – 1.41 | 0.452 |
| Complete case analysis ^b^ | 1.02 | 0.40 – 2.64 | 0.962 |
| SAPS 3 model ^c^ | 0.77 | 0.45 – 1.31 | 0.338 |
| PSI IV/V only ^d^ | 0.83 | 0.47 – 1.46 | 0.510 |
| ED admission only ^e^ | 1.03 | 0.51 – 2.08 | 0.938 |
| Palliative care excluded ^f^ | 0.78 | 0.44 – 1.38 | 0.394 |

^*^ Noninvasive ventilation is the exposure and invasive mechanical ventilation is the comparison group

^a^ Results from model 4 of table 2 (main manuscript)

^b^ Complete case analysis (132/369 – 36 % of the whole sample)

^c^ Results from model 5 (adjusting for model 2 variables + SAPS 3)

^d^ Results from the same variables of model 4 (final model) after excluding patients with PSI scores lower than IV/V (344/369 – 93 % of the whole sample)

^e^ Results from the same variables of model 4 (final model) after excluding patients not admitted directly from the ED (225/369 – 61 % of the whole sample)

^f^ Results from the same variables of model 4 (final model), after excluding patients who had a decision of withholding or withdrawal of organ support in the first 24 hours (344/369 – 93 % of the whole sample)
