# Supplementary material for: Noninvasive ventilation in critically ill very old patients with pneumonia: A multicenter retrospective cohort study
Source: PLoS One. 2021 Jan 27;16(1):e0246072. doi: 10.1371/journal.pone.0246072 (PMC7840033; doi:10.1371/journal.pone.0246072)
Supplement: S3 Table — (DOCX) [file pone.0246072.s008.docx]

## S3 Table. As-treated analysis for the primary outcome.

| **Patient group** | **Odds ratio** | **95% CI** | **p-value** |
| --- | --- | --- | --- |
| Group 1 | 0.52 | 0.28 – 0.97 | 0.039 |
| Group 2 | 1.15 | 0.44 – 3.04 | 0.771 |
| Group 3 | 3.22 | 1.21 – 8.55 | 0.019 |

Group 1: NIV group patients’ who were never intubated throughout ICU stay.

Group 2: NIV group patients’ who were intubated within 24 hours of ICU admission.

Group 3: NIV group patients’ who were intubated after 24 hours of ICU admission.

The reference group were those patients who had IMV as first ventilatory support strategy.
