# Supplementary material for: Noninvasive ventilation in critically ill very old patients with pneumonia: A multicenter retrospective cohort study
Source: PLoS One. 2021 Jan 27;16(1):e0246072. doi: 10.1371/journal.pone.0246072 (PMC7840033; doi:10.1371/journal.pone.0246072)
Supplement: S1 File — (DOCX) [file pone.0246072.s009.docx]

# S1 File. Additional methods

In this session, we provide additional details about the methods. Since the logistic regression model in this manuscript is used to answer a causal question, a causal directed acyclic graph (DAG) was built (S1 Fig) to select the best set of covariates to address confounding and avoid collider-stratification bias ^1^. The directed acyclic graph was built with the online version of DAGitty ^2^.

## Modelling decisions

We evaluated variables from the DAG to assess the best functional form of including them in the model, especially continuous covariates. To do so, we considered the following issues:

1. Non-linear relationships could reduce the adjustment for confounding of the model. Therefore, we evaluated for non-linear relationships in the log-odds scale in different, but complementary ways: (1) including the variable in the model as an indicator variable and assessing its relationship with outcome with the margins command and deciding if the relationship was approximately linear by eye-balling the graph; (2) using likelihood ratio tests to compare the best functional form (example: categorization vs. continuous);
2. Truncation of discrete covariates due to a low number of observations at the right-tail of the distribution;
3. Different levels of a categorical covariate could be collapsed if the number of observations would be low and the variable would not have its clinical meaning withheld;

After considering these issues, variables were included in the model in the following functional forms:

- Age, BMI and SAPS 3 score were included in the respective models as continuous covariates.
- Non-respiratory SOFA score was included in the models as a continuous covariate, truncating the maximum non-respiratory SOFA score at 10 points.
- pH was included in the model as a binary covariate (< 7.3 or ≥ 7.3) to allow inclusion as an interaction term in the model while maintaining its relationship with outcome.
- P_a_o_2_/F_i_o_2_ ratio was included in the model as a binary covariate (< 150 or ≥ 150) to allow inclusion as an interaction term in the model while maintaining its relationship with outcome and respecting previous descriptions where this cut-off was used to detect higher risk of both death and failure of non-invasive ventilation ^3^.
- P_a_co_2_ was included in the model as a binary covariate (< 50 or ≥ 50) respecting previous studies using this cut-off.
- Functional status was collapsed and included in the model as a binary covariate (restrict / bedridden vs. non-bedridden) to allow inclusion as an interaction term in the model while maintaining its relationship with outcome.

## Missing data handling

We evaluated the variables to be included in the model for missing data. During the data management process, we checked all variables to assess consistency. Impossible values were considered missing when they could not be checked against other variables in the dataset. Variables with missing data were the following: SOFA score, BMI, and variables related to arterial blood gases (ABG). P_a_co_2_ and pH were imputed from venous blood gases, when available. After data cleaning, we came to the values observed in S1 Table regarding missing data.

To impute these variables, we first decided whether imputation was acceptable. We assumed the data to be missing at random (MAR). This assumption is not testable by the data itself. It assumes that the observed values can be used to explain data missingness and help predict what the missing value would be. To do so, a method such as multiple imputations (MI) is recommended. Briefly, during MI, a number of datasets is created imputing missing data based on the variables included in an imputation model. After the datasets are created, in the analysis stage, the standard errors are adjusted to account for the multiply imputed datasets by using Rubin’s rules: this allows that uncertainty brought by missing data is accounted for in the outcome model, therefore leading to less biased estimates than complete case analysis or other forms of imputation.

After assuming MAR and deciding to do MI, we decided to use 20 datasets for MI. Another important decision is related to the imputation model. We included all covariates, the outcome variable and all interaction terms that would be tested in the outcome model. Furthermore, we included auxiliary variables, which make the assumption of MAR more reasonable. They were the hospital where the patient was admitted, the year of ICU admission, the Charlson comorbidity index and SAPS 3 score.

We used the MI with chained equations (MICE) algorithm using predictive mean matching (PMM) to impute the data. PMM is a flexible model that allows for nonlinearities and leads to easier convergence of the model ^4^.

We did sensitivity analyses to evaluate robustness of the MICE model: first, we did a complete case analysis (which was expected to have very low power to detect any differences between groups); second, we did an analysis using SAPS 3 in the model instead of including other variables with more missing data, with the rationale that SAPS 3 includes pH, P_a_o_2_/F_i_o_2_ and components of the SOFA score in its determination. As described in the manuscript, the results did not differ for the sensitivity analyses.

# References

1. Lederer DJ, Bell SC, Branson RD, et al. Control of Confounding and Reporting of Results in Causal Inference Studies. Guidance for Authors from Editors of Respiratory, Sleep, and Critical Care Journals. *Ann Am Thorac Soc.* 2019;16(1):22-28.

2. Textor J, van der Zander B, Gilthorpe MS, Liskiewicz M, Ellison GT. Robust causal inference using directed acyclic graphs: the R package 'dagitty'. *Int J Epidemiol.* 2016;45(6):1887-1894.

3. Antonelli M, Conti G, Moro ML, et al. Predictors of failure of noninvasive positive pressure ventilation in patients with acute hypoxemic respiratory failure: a multi-center study. *Intensive care medicine.* 2001;27(11):1718-1728.

4. White IR, Royston P, Wood AM. Multiple imputation using chained equations: Issues and guidance for practice. *Stat Med.* 2011;30(4):377-399.
